# Opinion: Applications of and Barriers to the Use of Biomimicry towards a Sustainable Architectural, Engineering and Construction Industry Based on Interviews from Experts and Practitioners in the Field

**DOI:** 10.3390/biomimetics9080470

**Published:** 2024-08-03

**Authors:** Rory V. Jones, Alba Fuertes, Roman Scherer, Derek Clements-Croome

**Affiliations:** 1 School of the Built Environment, University of Reading, Reading RG6 6UR, UK; a.fuertes@reading.ac.uk (A.F.); d.j.clements-croome@reading.ac.uk (D.C.-C.); 2 Scherer Architekten, 4410 Liestal, Switzerland; r.scherer@scherer-arch.ch

**Keywords:** biomimicry, sustainability, built environment industry, sustainable buildings, semi-structured interviews, practitioners

## Abstract

Biomimicry creates designs inspired by nature and uses ecological benchmarks to assess their sustainability. It is believed that biomimicry can help society produce and consume in more sustainable ways, as well as address some of the key challenges facing the world today. However, research into the applications of and possible barriers to using biomimicry for creating more sustainable Architectural, Engineering and Construction (AEC) projects is still limited. This paper addresses this gap by undertaking and analysing twelve semi-structured interviews with leading global experts and practitioners in the field of biomimicry as applied to the built environment industry. The study identifies substantial potential in the use of biomimicry in AEC projects, including the following: adopting circular approaches; enhancing interactions between human and natural infrastructure; optimising material and energy use; recycling and re-use of materials; reducing time and costs; plus more collaborative and interdisciplinary working. However, a wide range of multifaceted barriers also exist that are currently hindering the exploration and exploitation of this potential, including the following: lack of knowledge; insufficient research and testing at the scale of AEC projects; fragmentation, poor communication and traditional nature of the industry; perception of high risks and costs; as well as outdated and unsuitable legislation and planning processes.

## 1. Introduction

The terms biomimetics, devised by Otto Schmitt in the 1950s, and bionics, first mentioned by Jack Steele in 1960 [1], laid the foundations for the term biomimicry. Biomimicry, biomimetics and bionics all represent biologically informed disciplines for the purpose of innovation to address human challenges [2]. A useful review of definitions is provided by Verbrugghe et al. [3]. Biomimetics is the creation of materials or products inspired by the structure and function of living things [4], and bionics is the design of engineering systems based on biological systems [5]. According to Benyus [6], however, the goal of biomimicry is to create innovative sustainable solutions mimicking nature. Biomimicry, as a science, creates designs inspired by nature in terms of form, processes and systems and uses ecological benchmarks to assess their sustainability [6]. It is believed that the study of biomimicry might reveal promising opportunities for society to produce and consume in more sustainable ways [7]. Biomimicry therefore presents a potential approach to address and mitigate some of the main challenges facing the world today, such as climate change, environmental and ecological damage, issues with energy supply and demand, etc. [8].

The idea of design inspired by or mimicking nature is not new, as evidenced by Leonardo da Vinci’s (1452–1519) flying machine or ornithopter, which was inspired by bats, kites and birds [9], or Filippo Brunelleschi’s construction technique used for the Duomo in Florence in 1436 [7]. There are many published books and articles describing applications of nature in architecture and engineering (see [10,11,12]). For example, Clements-Croome [13] discusses a range of cases of how, by observing nature, it is possible to discover new ideas for the Architectural, Engineering and Construction (AEC) industry, such as composites inspired by mollusc shell nacre, suspension cables inspired by spider dragline silk, adhesives inspired by the setae on the toes of geckoes, etc.

The roots of biomimicry, as a science for creating more sustainable solutions for the AEC sector, were laid centuries ago, but its application in architecture and engineering matured in the 1950s and 1960s and more recently with the work of Benyus [14]. According to Verbrugghe et al. [3], the field of biomimicry however remains quite abstract, requiring more research to uncover its specifics, true meaning and potential. They also note that whilst academic literature on biomimicry is rapidly growing (1011 publications between 2019–2022 as opposed to only 17 between 1997–2000), its use in the AEC sector is not widespread and still lacks a generalised and shared methodology, and there remains a need to understand experts’ and practitioners’ perspectives on the potential applications of and barriers to this promising field encountered in real built projects.

From the academic literature, Jamei and Vrcelj [15] provide a useful review of potential opportunities for the use of biomimicry in architecture and building engineering, including choice of materials, structural design and behaviour, the design and operation of mechanical and electrical services, and fabric envelope design. Kapsali [16] presents examples of how natural processes and phenomena have informed real-world design and discusses what might be possible if designers in the AEC industry use nature as a starting point for their research and inspiration. Gorb and Gorb [17] discuss how challenges in architecture such as multi-functionality and sustainability have been solved by insects in their evolution in ways that could be applied in the AEC sector.

A framework for applying biomimicry to a design or architectural problem has also been proposed by Zari [18,19]. The framework comprises three levels at which biomimicry can inform architecture: organism level (imitation of nature’s form, shape and structure); behaviour level (imitation of natural processes); and ecosystem level (imitation of how organisms interact at an ecosystem scale). At each level, biomimicry presents five avenues for mimicking nature, in terms of applying look and form, materials, how it is constructed, how it works, and what it is capable of doing. Ilieva et al. [20] developed a further classification system, the Biomimicry for Sustainability framework, for assessing biomimicry projects based on their impact in terms of sustainability using two dimensions. The first encompasses the scope for mimicking nature in relation to sustainability and the second describes whether the mimesis is fixed or flexible.

One approach towards understanding the potential of biomimicry has been to apply theoretical frameworks like Zari [18,19] and Ilieva et al. [20] to exemplary built projects [21]. However, it should be noted that built building case studies using biomimicry are still scarce [21], particularly for large-scale projects where cost-effectiveness is hard to demonstrate. Verbrugghe et al. [3] and Cruz and Hubert [22] provide two valuable papers describing and analysing a range of real-world biomimicry projects using theoretical frameworks.

**Figure 1:**
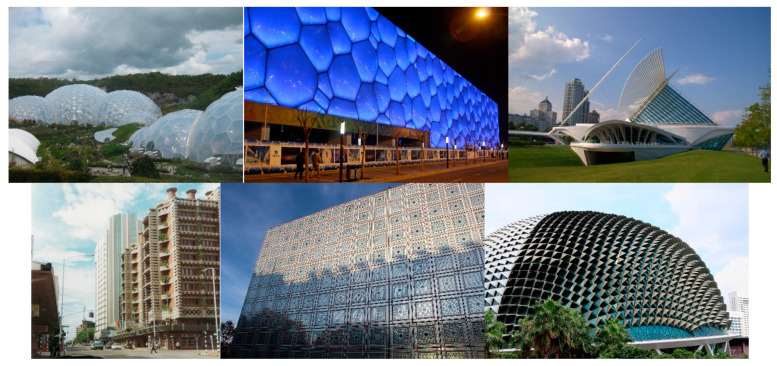
Architectural projects using biomimicry. From left to right (**Top**): Eden Project [24], National Aquatics Center [25], and Quadracci Pavilion [26]. From left to right (**Bottom**): Eastgate Centre [27], Arab World Institute [28], and Esplanade Theatre [29].

Another approach is analysing real architectural projects from around the world that have implemented biomimicry such as The Eden Project, UK, which uses a lightweight Ethylene Tetrafluoroethylene (ETFE) structure to cover a wide area mimicking soap bubbles and pollen grains; the National Aquatics Center (Water Cube), Beijing, also inspired by soap bubbles and similarly utilizing a lightweight, transparent and robust ETFE structure; Quadracci Pavilion, Wisconsin, with a kinetic shading system, the Burke Brise Soleil, which was derived from the form of a bird’s wing; Eastgate Centre, Zimbabwe, which incorporates a natural ventilation cooling system inspired by termite mounds; Arab World Institute, France, with light-responsive façade modules that open and close in a manner akin to the contraction and dilation of the iris of an eye; and Esplanade Theatre, Singapore, with a responsive façade that adjusts to the sun’s angle to provide shading inspired by the durian fruit (Figure 1). For more information on these and other exemplary projects, see Verbrugghe et al. [3] and Radwan and Arch [23].

The current paper utilises an alternative methodology of qualitative semi-structured interviews to investigate the potential applications of and barriers to the implementation of biomimicry in the AEC industry. To the authors’ knowledge, this methodological approach is not currently present in the literature on biomimicry. Furthermore, the paper also responds to a clear gap in the literature discussed above: there is a current need to collect and analyse the views and opinions of experts and practitioners working on real-world AEC projects using biomimicry to gain a deeper understanding of its potential applications and barriers to its use.

## 2. Materials and Methods

The study adopted a qualitative approach to data collection. Semi-structured interviews were undertaken with 12 leading global experts and practitioners in the field of biomimicry. The participants were selected due to their extensive professional experience in the field of biomimicry and the built environment and represented a diverse range of AEC professionals from Austria, Denmark, Germany, Italy, Switzerland, UK and USA (Table 1).

The snowball sampling technique [30] was used, whereby the initial recruited experts and practitioners were then asked to provide the contact details of other experts and practitioners in the field to participate. The final sample of 12 participants recruited covered a range of disciplines from the sector, including architecture, engineering and academia. The research was approved by the ethics committee of the University of Plymouth and informed consent was obtained from participants before data collection commenced.

The interviews took between 45 and 90 min to complete and included standardised, open-ended questions for all participants, as well as follow-up and personalised questions. The questions aimed to understand the participants’ experience, knowledge and views of the potential applications of and barriers to the implementation of biomimicry in the AEC industry. As the researcher was bilingual in English and German, interviews were conducted in English or German, depending on the participants’ preferred language. The main standardised questions asked of all participants were the following: (1) How would you define biomimicry? (2) How can biomimicry be used to increase the sustainability of Architecture, Engineering and Construction projects? and (3) What are the potential barriers to using biomimicry in Architecture, Engineering and Construction projects and how can these be overcome?

The interviews were conducted over Zoom or Webex and were audio-recorded and transcribed. A thematic analysis framework [31] was used for the interviews as it provided a flexible approach for the analysis of the qualitative data [32] with the key ideas cited by participants coded and then organised into broader themes [33,34].

## 3. Results

### 3.1. Definition of Biomimicry

Participants 1 and 4 acknowledged the sometimes ambiguous nature of the concept of biomimicry stating that ‘Everybody’s got his own idea and as you talk to different people, everybody will provide you with a slightly different answer’ (Participant 1) and ‘There are very different approaches. There are many interpretations of what biomimicry is and therefore it has to be kept within the framework and always clearly convey what you mean by the term in your own research work’ (Participant 4). Participant 9 also added ‘There is many words like biomimicry, biomimetics, bionics, bio-inspired design and so on which are often used interchangeably but have distinct differences’.

Participant 1 defined the term as ‘The idea of looking at biological design in a wider context, not just the materials but the functionality of the systems and then try to see if parts of it could be abstracted and translated into technical solutions’. Participant 2 provided a similar definition: ‘The abstraction of design from nature. It’s looking at the mechanisms in nature that may be adapted’, and added ‘It’s not copying, it’s studying how nature does something to achieve a result. For the example of transpiration, you can look at the way fluids move around through stems and leaves–you find that the way fluids are conducted consumes a minimum of energy. From that you can derive transport algorithms that are much more efficient’.

Participant 3 described biomimicry as ‘A process at the beginning of which a biological material, system or organism is examined very intensively in order to capture its underlying structures, properties or functional relationships. (…) it needs an abstraction process because the biological system usually is multifunctional, while the technical application usually only requires one function. (…) the next step is the material development and engineering by identifying which similar materials I can apply the principle seen in the abstraction process’. Participant 4 stated ‘For me, it (biomimicry) is exploring biological principles and applying them to mechanical or structural problems’. And for Participant 12, ‘Biomimetics is a creative reinvention, inspired by role models from nature. This takes many iterative, creative steps to transfer the properties of an organism into a technical product’. Participant 7 summarised it as ‘It’s essentially integrating biology and engineering in some way but when you boil it all down it’s basically another way of designing’.

Participant 5 addressed the concept of biomimicry by comparing it to the terms bionics and biomimetics: ‘The biomimicry group that is most prominent in the USA comes from the biology side and ties the concept of ecology and sustainability (…) Bionics and biomimetics have proven to be pure innovation methods and do not offer an evaluation of sustainability. That means you can produce a very toxic product even with a biomimetic approach. The element of sustainability only comes into play at the end of product development’. Participants 8 and 11 also commented on the association of the term biomimicry with sustainability. Participant 8 defined the term as ‘A deliberate quest or search in nature for something that can solve a challenge (…) I personally use biomimicry as a search for functions to solve a problem’ and added ‘the requirement of sustainability doesn’t have to be a killer criterion’. In contrast, Participant 11 made reference to Janine Benyus’s definition and defined biomimicry as ‘An emulation of nature’s form, functions and process to create more sustainable human environments’.

Participant 9 defined biomimicry as ‘the conscious emulations of nature’s adaptations to solve human challenges’.

Participant 6 described biomimicry as ‘the mimicking of ecosystem services of a local ecology, meaning it’s not mimicking an individual plant or animal but it’s the mimicking of how the entire ecosystem works’.

### 3.2. Applications of Biomimicry to Achieve Sustainable AEC Projects

All participants agreed that there are many potential ways to use biomimicry to increase sustainability in architecture, engineering and construction projects. These opportunities could broadly be categorised using the classic three pillars of sustainability: environmental, economic and social.

#### 3.2.1. Environmental

First, Participant 9 explained the overarching concept as to how nature can create more sustainable AEC projects is that ‘nature has evolved as a circular economy, therefore there is no waste, only resources. Many current cities have been designed with a very linear metabolism consuming resources, producing waste and disposing of them. There’s no circularity to it’. Therefore, one way sustainability can be increased is by ‘looking at the ecosystem level of design and seeing how ecosystems perform. And therefore learn how we design out the linear metabolism’ for AEC projects.

Participant 6 agreed, and explained that they have started ‘working on a different scale to apply biomimicry–the ecosystem scale where we mimic ecosystem services’. Participant 6 defined this approach ‘as the mimicking of ecosystem services of a local ecology, meaning it’s not mimicking an individual plant or animal but it’s the mimicking of how the entire ecosystem works’. This approach allows an AEC project to become ‘a regenerative design where we step past net zero and get closer to a restoration of the ecosystem services that the ecology of this place used to provide’.

Participant 12 added that an ecosystem approach embedded in the urban planning process can also be used to improve sustainability: ‘one should see buildings in a wider context in interaction with the environment. This includes the placement, orientation, and massing of the buildings, which, for example, could prevent the draught into a block or neighbourhood, which leads to elevated temperatures. In this way, biomimicry solutions can be optimally used in the regulation and formulation of urban planning interventions’. Participant 12 explained that this has been made possible ‘due to new production and simulation methods’.

Participant 5 stated that ‘a part of biomimicry is moving away from the abstract transfer of principles towards more integration of living organisms into the architectural structure and its surroundings’. Using this integration concept, sustainability can be improved as it ‘generates space for humans and local ecosystems alike or prevents the destruction of the latter in the first place’. Participant 2 took this thought further and suggested that ‘we should think about buildings as organisms rather than static blocks of material’ and Participant 12 agreed, saying ‘this means that the house has life like properties’.

Participant 8 said that ‘one of the ways in which nature is sustainable, and in which I think we should learn more from nature, is the way it uses a very limited number of elements to build everything. This also allows it to break them apart easily and reuse them to form a circular economy’. This idea can be applied in the production and eventual demolition of individual buildings. Participant 7 explained that we should use solutions in the AEC industry ‘that we will be able to grind up and reuse it, which you can’t do with brick or with any sorts of mortar’. Participant 7 pointed out that this biomimicry principle is however not new and has been used for a very long time and ‘you find this in a number of vernacular’ AEC projects.

Participant 2 said that another route to sustainability using biomimicry is optimisation, as ‘nature optimises its use of energy and materials’. Participant 7 reinforced this argument with ‘in technology, material is cheap and design is expensive’ but ‘in nature, material is expensive and design is cheap’. Therefore, biomimicry-informed AEC projects can use this principle to find ways to avoid excessive use and waste of materials. Participant 4 explained that they had undertaken a study which ‘showed that the biomimetic end product was more sustainable than a traditional built structure due to its resource efficiency and use of local materials’ and Participant 12 added that this is also ‘good for the carbon foot-print’ of the building, too.

#### 3.2.2. Economic

As previously mentioned, the use of biomimicry in AEC projects can optimise the use of materials and energy through more efficient design, thus resulting in monetary cost reductions during both the construction process and lifetime operation of the building. Participant 12 explained that ‘in the field of lightweight construction with fibre composites, solutions with concrete can save 20–30% mass’; also, ‘one could well save material in places where they are not critical’.

Participant 1 also explained that the use of biomimicry can create additional energy and cost savings for projects, as ‘The more we can delegate functionality to the material system, the less electronics we’re going to need in order to control the behaviour–the more we can integrate these functions at a lower level, the less we have to add in terms of electronics and computing power’. In other words, the better the passive architectural design, the less energy consumed by the active building services systems. Participant 1 further stated that to minimise energy consumption ‘we need to increase the responsiveness of our buildings to make them cope with the variability of the environment’ and to achieve this, more sensors are needed in buildings, such as those we can find in nature which are ‘very simple design wise but incredibly smart. They are very cheap energy wise and therefore you can afford to have lots’.

Furthermore, another economically significant benefit of biomimicry is the circularity concept outlined earlier, whereby the recyclability of materials is considered for projects. Participant 7 explained that recycling materials will ‘reduce material production costs and be more energy efficient, as long as the recycling process does not need more energy than the mining of new material’.

Participant 3 also stated that reduced energy use and waste of materials can also be achieved by using principles of biomimicry for the processing of composite materials such as wood used in projects. Participant 3 explained how they use the properties of wood to layer and change the form to a desired shape without external forces: ‘The active component eliminates the requirement for additional technology which reduces energy consumption and the process step eliminates the need for mechanical forces to bend the material which again reduces energy consumption and offers a gentler treatment of the material’. Participant 7 gave another example and explained that ‘one of the reasons why design is cheap in nature is that a lot of the materials assemble themselves’.

Participant 12 explained that by understanding that biological structures are ‘usually never optimal, but optimised or good enough but with as little material and energy expenditures as possible’, AEC projects could use this approach to reduce the time and costs of their architectural and engineering design processes. Although, Participant 12 noted that ‘making something good enough as a goal for the application is a thought process that engineers and architects do not find easy’.

Additionally, Participant 2 explained that ‘Nature is very durable, consistent and wears well’ and that there could therefore be potential financial savings from reduced maintenance and replacement of building components during a building’s lifespan.

#### 3.2.3. Social

Many participants agreed that a biomimicry-informed AEC project encourages more collaborative, interdisciplinary thinking and working. Participant 11 explained that ‘in nature everything is interconnected and relies on each other and I think interconnection and interdisciplinarity is what we need to aim for if we even want to attempt to use biomimicry’. Similarly, Participant 2 saw a clear chance in this approach and indicated a need for a profound change in the way AEC professionals work together: ‘biomimicry encourages you to think multilaterally’ (…) ‘I believe you’ve got to have a meeting of minds. In nature it might be less obvious than people coming together but it’s constantly happening. Nature is united and we are not’. Nature suggests that the current approaches used in the AEC industry are ‘broken apart and there’s no connectivity’ which is in contrast to nature where ‘connectivity is good’ (Participant 2).

In addition to collaboration between project professionals, biomimicry also inspires more and better synchronisation between people and nature. Participant 5 described that a ‘branch of biomimicry is looking at the creation of combined habitats for humans and nature, which is an element of sustainability where you can achieve a healthy environment for all participants’. The idea of ‘rediscovery’ (Participant 12) of the natural world and its relationship with AEC projects was expressed by nearly half of the participants (Participants 2, 5, 9, 11, and 12).

Another aspect that biomimicry can teach is that ‘in the natural world, things are a little bit more constant. They do change, but it’s a gradual change’ (Participant 2), which is in contrast to humans where ‘society and expectations are changing rapidly’ (Participant 2). Therefore, one of the possible lessons from biomimicry is to more carefully consider the need for the AEC technologies to be implemented; this was explained by Participant 11: ‘before we create new technologies, we might need to take a step back and first think about whether our actions are needed’.

#### 3.2.4. Dissent

Despite the range of perceived sustainability benefits for the environment, economy and society mentioned, many participants also emphasised that ‘it is not a straight equation that biomimicry leads to a sustainable design or products’ (Participant 11). Participant 12 elaborated that this was ‘a major error of thought… it is a false conclusion that biomimetic or bionic solutions are inherently sustainable. This is wrong and sustainability must be tested individually for each element or construct. The reason is that nature is not sustainable’. Participant 8 and Participant 10 supported this view ‘there is no law that only because something is derived from nature it is going to be sustainable’ (Participant 10) and ‘nature is sustainable in many ways, but it does not necessarily match our understanding of sustainability’ (Participant 8). Participant 12 explained that ‘sustainability is a human-made concept in which you want to preserve resources for the next generations. This is not how evolution works’ in nature.

Furthermore, Participant 5 specified that there is a regional difference in the definition of biomimicry: ‘In the US the most prominent group of biomimicry professionals use sustainability as a selling point for biomimicry and it is a separate part of the early evaluation process. In Europe, biomimicry or bionics are mostly understood as a pure method of innovation. This means that you can develop a product with a biomimetic approach which can be very toxic. The element of sustainability is the main difference between biomimicry (USA) and biomimetics (Europe)’.

### 3.3. Barriers to the Use of Biomimicry to Achieve Sustainable Architecture, Engineering and Construction Projects

#### 3.3.1. Lack of Knowledge and Research Gaps

A general lack of knowledge was stated as a barrier to adoption of biomimicry: ‘a big reason people don’t talk about new ways of doing things more is because they don’t know about it’ (Participant 9), and although it was noted that the concept of biomimicry has been around for some time, ‘there is a myriad of things in many different fields where you can discover something new almost weekly which is a nice thing about biomimicry’ (Participant 2). However, this means that there is still a lot of new and ongoing research in the field and because of that ‘at the moment you’ll find it very difficult to use biomimicry because nobody has really done that analysis very well’ (Participant 7). Participant 6 agreed, and stated ‘you have to get the dataset first. Obviously, the research effort it takes to gather and analyse the data is expensive and takes a lot of hours’. Participant 5 concurred and explained that ‘in order to gain validity and understanding of the technology, one must have not only descriptive, but also causal knowledge. We need much more basic research in order to be able to take this step’. Participant 12 saw a need for ‘basic research in biology, engineering, architecture and materials science’.

Nine out of the twelve participants indicated that there was also a need for more strategic ways of finding and researching new technologies from nature for use in AEC projects, which contrasts with the ‘rather random observations like Velcro for example or the Lotus effect’ (Participant 1). Participant 5 explained that ‘In order to enable a top-down development, a basis must be created where you have the people that you need at your hand. The biologists, bio-physicists, etc. who can feed the basic research. Otherwise, the R & D takes far too long, and costs are too much’.

Participant 7 summed all this up with the blunt statement that ‘the barrier is essentially ignorance, one of the usual suspects’.

#### 3.3.2. Nature of the AEC Industry

Over half of the participants thought that the inherently slow pace of the AEC industry and lower proportion of highly educated people compared to other sectors plays a role in the slow adoption of biomimicry. Participant 2 stated that ‘construction is a very slow-moving industry and very traditional’ and ‘it is not the same as the automotive, aerospace or nuclear industries where they have a higher proportion of highly educated people’. Participant 9 put a number on the time lag and said that ‘architecture is always 20 years behind most other industries. We are late adopters’. This statement was supported by Participant 11 who said ‘architecture is a slow evolving field’.

Participant 9 provided a possible explanation, stating that ‘the problem is that architecture is an amalgamator of many different technologies’, therefore biomimicry is just one of many possible solutions. A practical explanation was given by Participant 10 who pointed out that ‘it is especially difficult in architecture to test new systems in real world scenarios due to the size of it. This makes the jump from research to a market launch extremely difficult’; as a result, demonstrating the benefits of biomimicry is challenging.

Participant 8 also explained that ‘one barrier is that we have a tradition of doing things or building things a certain way. Our whole economy is built on concrete and alternative options are often overlooked simply because we have the tradition, the skilled labour and dimensions bound to concrete’. Participant 11 supported this thought and pointed out that ‘we want new or better solutions within our traditional and comfortable toolbox and are reluctant to give up old ways of thinking and try new unfamiliar tools’.

#### 3.3.3. Industry Fragmentation and Poor Communication

Uptake of biomimicry in AEC projects suffers from the ‘fragmentation of the industry and silo thinking’ (Participant 2) and a ‘lack of a common language’ (Participant 10) between professionals. The latter was considered particularly pertinent in relation to biomimicry with 8 participants stating that ‘there are many different approaches to the understanding of what biomimicry is, depending on who you ask’ (Participant 5) and ‘everybody’s got their own idea and as you talk to different people, everybody will provide you with a slightly different answer’ (Participant 1).

Participant 5 and Participant 2 agreed, explaining that many mistakes happen ‘because of misunderstandings, different language use, different interpretation of things and different mindsets’ (Participant 2). Participant 10 stated that this leads to ‘missing out on many biological solutions because architects and engineers cannot express themselves well enough to the biologists to convey what exactly they are trying to solve. Additionally, even the biologists are sometimes so specialised that they overlook common denominators’. Participant 1 explained ‘that people often design materials without exactly knowing for what it is going to be used. This is where the integration and communication between disciplines is important. Because if people knew that there is a specific demand in an architectural context, they would start putting their heads together and produce something that can help and solve that problem’.

Even though there seems to be a communication problem between the AEC professions and professionals, Participant 1 said that ‘I don’t think it’s a major one. One does not want to get bogged down by semantics. I think so long as people understand that there is a potential of a technology transfer from the biological world to a technical world the final details of how you see it and how you interpret it are of little importance’. Participant 10, however, pointed out that ‘even though we develop a common language over time it takes a very long time to establish one’. Participant 2 allocated part of the problem to ‘the education in the building industry’ which ‘is very broken up into pieces’ and said that ‘in the end, everyone needs to come together and for that we need to bridge the language and cultural barriers’.

#### 3.3.4. Risk and Cost

Perceived risk and the financial implications of managing the risk were seen as barriers to the uptake of biomimicry in the AEC industry. Participant 12 described that ‘Building a house is a costly business that an average income earner only does once in a lifetime’ and therefore ‘few are willing to take an extra risk without any support’. This was also seen to equally apply to bigger investors, as Participant 9 explained that ‘with any new product you need to prove that it meets all the fire regulations, acoustics, insulation level etc., this all takes time and money with the potential outcome that the product actually doesn’t comply. This is a massive risk that a lot of investors are not willing or can’t take because their model and margins don’t allow for the R&D unless they are especially designed that way’. Participant 9 stated that ‘biomimicry related projects are not for every client and most clients don’t want to be first or second, they don’t want to be the ones who are taking the risk’.

However, Participant 2 discouraged the thought that nature-inspired solutions are intrinsically risky by explaining ‘It can give us confidence, because nature is what it is and it’s been consistent over a long period of time, so it doesn’t carry the [same level of] risk as some of the new [non nature-inspired] inventions. People are sometimes reluctant to pay into a new invention because they don’t know if it will work. Well, with nature you’ve got a lot of evidence’.

Participant 7 added that the use of biomimicry in AEC projects was not only a financial risk but a personal risk for the scientists developing the technologies and products for fields outside their direct expertise, such as architecture: ‘unfortunately, most scientists are frightened of moving out of their general area’. This often means that larger design teams with a wider range of expertise are needed for AEC projects ‘around the design table from day one’ (Participant 9) with the negative effect that ‘most clients would look at you and say, are you mad, how much do you want me to spend on professionals’ (Participant 9).

Participant 5 and Participant 10 suggested that one way to show that biomimicry solutions do not carry such high risk is ‘big one-off projects and expos in which a lot of money is put in without a realistic claim to profit and are extremely important to advance the research and show that something is possible. Once proven, an adaptation can take place at lower levels’ (Participant 5). Another benefit of these one-off, expo-type projects is that it ‘shows the public not only that such a thing exists but also that is works’ (Participant 10). Participant 12 agreed ‘the more projects we can implement that work well, the greater the acceptance and chance that a city will tackle such a project. I hope that such a multiplier effect can set the avalanche in motion’.

Multiple participants agreed that perception of cost is something that needed to change. Participant 2 said ‘People’s perception about cost stops the process. If you say anything unusual, they might say: Oh yes very interesting, fascinating, but very expensive. I then say: but how do you know that? You don’t know that it is expensive. There’s a lot assumptions’. Participant 1 also said that ‘if we also start to pay for things in terms of energy used or carbon dioxide produced, as opposed to pounds, things would start to shift’ and Participant 9 shared an optimistic view, stating that ‘I do think that the mindset is changing quite rapidly now, and people are becoming aware that the return on investment isn’t actually only financial but also carbon savings and the impact on the environment might also need to be considered’. This argument was also supported by Participant 12: ‘the problem with these solutions is not so much money, but a willingness to rethink in a flexible manner’.

#### 3.3.5. Time

In relation to time, Participant 11 indicated that ‘we need to change our perception of time when it comes to key innovations, they do take a long time’ and it was thought that this certainly applied to the use of biomimicry in the AEC industry. Participant 2 agreed, saying ‘a lot has to do with our expectancies’ of time.

#### 3.3.6. Regulations and Planning

10 participants regarded the current regulatory system in the AEC industry as one of the major challenges. This was common in all the countries represented in the study. Participant 1 mentioned that ‘The problem of architecture, and to some extent the problem with other engineering sectors, is to change the frame of mind of the regulatory bodies. Certain things have been locked in building regulations or have evolved on a completely different path and if you want to use a new path it is almost impossible to fit in with the existing regulations. This means there is a big effort to be made in relation to convincing the regulatory bodies that things are possible’.

Participant 5 agreed, and expanded that ‘another obstacle is the material standards, which are not designed to evaluate biological materials. For example, the multifunctionality of a material is not taken into account because it is only evaluated in one way and then compared with other materials’. This results in ‘even things that are very logical and natural to a researcher might not be included in the building codes’ (Participant 11) and that ‘even though you find amazing solutions that can reduce cost and increase sustainability there is a bottle neck in the system which is the regulatory system’ (Participant 9).

Participant 12 indicated that ‘there are certain limits and standards that should not be lowered’ but stated that ‘a lot of them should be customisable and relative to the environment we build in. The strict guidelines in the building law certainly do not help to make progress in these areas’. Participant 12 further explained that ‘through this reluctance to change, I see repeatedly wonderful opportunities being squashed, which would need a little more effort and brain lard in the planning stage, but fail, because of the unwillingness of bureaucratic units’. Participant 12 went as far as saying that ‘the biggest problem is still over-bureaucratisation, which suffocates rather than encourages courage. This also brings the fear of being sued, which a smaller architecture office or engineering office simply cannot afford’.

Participant 6 gave an illustrative example from New York where they undertook AEC projects explaining that ‘if the fire department doesn’t like it, it doesn’t happen in NYC. Unfortunately, this influence of what the fire department likes or doesn’t like is influenced by certain unions as well. This means that you can’t build a timber structure in NYC because the fire department won’t allow you and they won’t allow you because they are heavily influenced by the concrete and steel unions’.

Participant 1 believed that in some cases possible safety concerns around new biomimicry solutions were borne out of a delay in updating the regulatory process rather than an actual safety risk: ‘there is no particular reason why a building made in one way should be more of less safe than a standard one. The difference is that the safety regulations of the standard one have been integrated in the design codes whereas the new way of designing have not’.

## 4. Discussion

This paper has identified that both the applications of and barriers to the use of biomimicry in the AEC industry are multifaceted and in no short supply. Overall, the results indicated a broad range of potential opportunities for AEC projects to improve their sustainability using biomimicry, and these were categorised using the three classic pillars of sustainability: environmental, economic and social. The potential applications identified in this paper largely concur with those expressed in Jamei and Vrcelj [15], including material choice, structural design and behaviour, design and operation of the mechanical and electrical services, as well as fabric envelope design.

The circularity of natural ecosystems was seen as a key biomimicry concept that could be applied to AEC projects, whereby the often linear metabolism of resource consumption, waste generation and disposal is addressed for buildings and cities, and instead waste becomes a resource. At the ecosystem scale, there were also lessons for urban planning to improve how new projects interact with existing buildings and infrastructure from a passive design perspective, as well as with nature itself, moving towards regenerative designs that would restore natural ecosystems and generate space for both humans and nature. At this moment, the ecosystem scale of biomimicry is potentially the most easily applicable to the field of AEC, as the evaluation of these parameters could form part of a traditional site analysis and be implemented using existing technologies and practices. This finding reflects the research expressed by Zari [18,19] for the ecosystem-scale level of their biomimicry framework.

Optimising the use of material and energy for the production and operation of buildings and recycling materials at eventual demolition were also suggested as aspects that could be adopted from nature. Optimisation and re-use of materials were also viewed as possible routes to reducing the economic costs of both the construction and operation of buildings. Applying system and structural optimisation to AEC projects, however, requires substantial computational analysis and digitalised fabrication methods, and whilst the current rapid rate of digitalisation of the AEC industry is evident, use of such technologies may not be commonplace for some time, especially among smaller, traditional companies in the sector. Although, this will likely be solved through the training of new AEC professionals.

Whilst optimisation is important, it was also clear that biological structures are never usually optimal, but instead, are just good enough. This modus operandi from nature could also be adopted by architects and engineers to reduce the time and cost of their design processes (Process: a series of actions or steps taken in order to achieve a particular end). Furthermore, an understanding that in nature everything is interconnected, suggests that AEC professionals may also need to work more collaboratively and interdisciplinarily in order to successfully apply biomimicry to their projects.

A range of barriers to the adoption of biomimicry in the AEC industry emerged in this study. It was clear that more basic research and data collection was needed to make biomimicry-based technologies available to a broader spectrum of professionals and the industry in general. It was clear from the experts and practitioners interviewed that with further research and development, there was significant potential in biomimicry for AEC projects, but before they could be applied a greater understanding of their impact on the environment and humans was necessary.

The need for greater interdisciplinarity, interconnection and clear communication (Communication: the imparting or exchanging of information by speaking, writing, or using some other medium) in the AEC industry was seen as an important key to unlock the potential of biomimicry. It was clear that the AEC industry, including the scientific community, was viewed as fragmented and lacked clear communication. This was borne out in multiple guises, from professionals’ personal fear of leaving their expert comfort zones to educational fragmentation leading to silo-thinking. The lack of connectivity between the different stakeholders limits the potential in the exploration of new technologies and creates barriers for the launch of solutions. These are aspects which have also been expressed in previous research [3,23] when analysing real-world projects from around the world that have implemented biomimicry.

A paradigm shift in the perception of costs and risks was considered necessary to accelerate the uptake of biomimicry in the AEC industry. It was evident that a more holistic consideration of costs, beyond solely financial, such as carbon costs, could shift evaluations of the benefits of nature-based solutions in a positive direction.

An important role for governments, regulators and policy makers was clear, as outdated and unsuitable legislation and planning processes currently form a bottleneck for both the development and entry of biomimicry-based technologies into the AEC industry.

## 5. Conclusions

This paper investigated the potential applications of and barriers to the implementation of biomimicry in order to increase sustainability in the AEC industry. Semi-structured interviews were conducted with 12 leading global experts and practitioners in the field of biomimicry. The study identified substantial potential in the use of biomimicry; however, a wide array of multifaceted barriers is currently hindering the exploration and exploitation of this potential.

The key applications of biomimicry to increase sustainability in AEC projects identified are the following:

- Adopting the circularity seen in natural ecosystems, where there is no waste, only resources, to design buildings and cities.
- Consideration of the possible interactions of new projects with existing buildings and infrastructure to improve passive design (e.g., air flows, natural light, etc.), thereby reducing energy use.
- Thinking about interactions of new projects with nature itself, and using regenerative design, to restore natural ecosystems and create space for humans and nature.
- Optimisation of material and energy use in the production and operation of buildings.
- Recycling and re-use of materials from the demolition of buildings at the end of life.
- Reduced time and costs associated with architectural and engineering design processes by adopting the ‘good enough’ approach of nature.
- More collaborative and interdisciplinary working using the principle that everything in nature is interconnected.

The key barriers to the use of biomimicry in the AEC industry identified are the following:

- Lack of knowledge about the potential applications.
- The benefits of solutions still need to be proven through further research and tested at the scale of AEC projects.
- Fragmentation, poor communication and the traditional nature of the AEC industry plays a role in slow adoption.
- Perceptions of the high risks and costs of using non-standard approaches and technologies.
- Outdated and unsuitable legislation and planning processes form a bottleneck for both development and market entry.

The findings of this study should be of interest to all stakeholders engaged in the development, application and regulation of biomimicry-based solutions in the AEC industry and may help them to overcome the multitude of barriers to a more effective use of biomimicry. However, it is clear that there is no quick-solve solution and that all stakeholders must become active in order to unlock the potential of biomimicry to increase sustainability in the architecture, engineering and construction sector.

### Limitations and Future Research

Five potential limitations deserve attention in the understanding of the results and future research. Firstly, the data was collected from a small sample of twelve participants. Whilst they were all very experienced in the biomimicry discipline, their views do not necessarily represent the full research and practice community and some applications or barriers may not be fully or adequately addressed; for example, biomimicry related to active energy, building services and energy systems. Such aspects would therefore warrant further future research targeting experts and practitioners specifically in these AEC sub-fields. In addition, the quotations from participants presented in the paper are reported exactly as they were stated in the interviews and these may oversimplify certain aspects or contain misconceptions. A larger number of interviewees, or the use of an alternative large-scale data collection method such as a questionnaire, should be considered for future research studies.

Secondly, a small number of countries are represented in this study. In order to overcome any possible contextual differences, future studies should consider conducting in-depth studies at national level.

Thirdly, this paper utilizes the environmental, economic and social analysis framework to primarily consider the impact of biomimicry on the design of buildings as well as building designers. A possible opportunity for further research, therefore, is to analyse the broader environmental, economic and social impacts of using biomimicry.

Fourthly, this paper, to the authors’ knowledge for the first time, has employed a qualitative research approach using semi-structured interviews. In order to continue to expand understanding of the potential applications of and barriers to the use of biomimicry in the AEC industry, researchers should consider the use of other quantitative and qualitative research methods.

Finally, as confirmed by the experts and practitioners in the field, there is some ambiguity in the use of the terms biomimicry, bionic, and biomimetics, and they are sometimes used interchangeably. Therefore, results need to be read with that in mind.

This paper has identified a wide range of multifaceted barriers to the exploration and exploitation of the substantial potential identified in the use of biomimicry in the AEC industry. It is therefore recommended that a possible next step for this research is to analyse potential solutions to overcome these obstacles effectively.

## Figures and Tables

**Table 1:** Descriptions of interview participants.

Participant	Country of Origin	Job Role and Background Relevant to Biomimicry in the AEC Industry. Years of Experience in the Field of Biomimicry
1	UK	Professor specialised in composite materials engineering. Author of over 100 academic papers and book contributions in the fields of biomechanics, biomimetics, smart materials and structures and bio-inspired technologies for architecture. 20+ years.
2	UK	Professor, President and board member of several industry bodies. Author of over 100 academic papers and book contributions in the field of intelligent buildings, biomimicry and biophilia. 20+ years.
3	UK	Professor specialised in the transfer of biological principles and mechanisms into technical applications. Author of over 100 academic papers in the field of intelligent materials. 20+ years.
4	Germany	Head of Research at leading university for computer-based design and manufacturing. Author of over 100 academic papers and book contributions in the field of biomimicry. 15+ years.
5	Austria	Chartered Architect and Research Fellow in biomimicry. Author of several academic papers and book contributions in the field of biomimicry. 20+ years.
6	USA	Co-founder of leading consulting firm in the field of sustainable design solutions for businesses, government and civil society. Works with Fortune 500 companies, universities, non-profit organisations, US military and international governments. 20+ years.
7	Switzerland	Professor specialised in biophysics and biomimicry. Author of over 100 academic papers and book contributions in the field of biomimicry. Consultant to leading architecture practices in biomimicry. 20+ years.
8	Denmark	Professor specialised in mechanical engineering, bio-inspiration and cross-disciplinary collaboration and knowledge transfer between domains. Author of over 100 academic papers in the field of biomimicry and computer aided design. 20+ years.
9	UK	Chartered Architect and design lead for large-scale biomimetic projects. Specialised in biomimicry and biophilia. 15+ years.
10	Germany	Chartered Architect and academic at leading university for biomimetics. Author of several academic papers and book contributions in the field of biomimicry. 15+ years.
11	Italy	Chartered Architect. Author of several academic papers as well as a book about biomimicry. 20+ years.
12	Germany	Professor specialised in biomimetics and biomechanics at leading university in the field of biomimicry. Author of over 100 academic papers and book contributions in the field of biomimicry. 20+ years.

## Data Availability

The authors confirm that the data supporting the findings of this study are available within the article.
